# PROBLEM-SOLVING THERAPY FOR HOSPICE FAMILY CAREGIVERS: DOES TIMING MATTER?

**DOI:** 10.1093/geroni/igae098.2749

**Published:** 2024-12-31

**Authors:** Oonjee Oh, Karla Washington, Debra Parker Oliver, George Demiris

**Affiliations:** University of Pennsylvania, Philadelphia, Pennsylvania, United States; Washington University in St. Louis, St. Louis, Missouri, United States; Washington University in St. Louis, St. Louis, Missouri, United States; University of Pennsylvania, Philadelphia, Pennsylvania, United States

## Abstract

Although problem-solving therapy (PST) shows promise in alleviating hospice caregivers’ distress, the best timing for its implementation (e.g., closer to admission or later) is unknown. Our aim was to compare changes in hospice caregivers’ depressive and anxiety symptoms based on whether they received PST during or after their transition to hospice. We conducted a secondary analysis using pre- and post-intervention data from a PST intervention labeled PISCES (Problem-solving Intervention to Support Caregivers in End-of-life care Settings). Among 218 hospice family caregivers who were actively caring for their loved one at baseline, 79 caregivers received PISCES face-to-face (F2F), 72 caregivers received PISCES via both F2F and videoconferencing (Hybrid), and 67 received an enhanced hybrid version of PISCES (PISCESplus), which additionally focused on recognizing positive aspects of caregiving following a positive reappraisal approach. The timing of the intervention was dichotomized into starting either during recent transition to hospice (within 14 days of hospice admission) or later. Caregivers who received PISCES F2F, whether during or after hospice transition, demonstrated improved depressive symptoms (p value 0.013 and < 0.001, respectively). PISCES Hybrid group did not show a significant change in their depressive symptoms at either timing. Within the PISCESplus group, only those who received the intervention later demonstrated a significant decrease in depressive symptoms (p value=0.043). Regarding anxiety symptoms, all caregivers experienced significant decrease, irrespective of the intervention type and its timing. Further efforts are needed to optimize the timing of PST in real-world hospice settings to increase its translatability and effectiveness.

